# Thyroid function status and its impact on clinical outcome in patients admitted to critical care

**DOI:** 10.12669/pjms.314.7497

**Published:** 2015

**Authors:** Faiza A. Qari

**Affiliations:** 1 Faiza A. Qari, FRCP, ABIM. Department of Internal Medicine, Faculty of Medicine, King Abdulaziz University, Jeddah, Saudi Arabia

**Keywords:** Medical intensive care unit, Surgical intensive care unit, Thyroid hormone profile, Mechanical ventilation

## Abstract

**Objective:**

To analyze alterations in thyroid function and the correlation between results of thyroid function test and mortality in medical and surgical intensive care unit (ICU) patients. It also aimed to evaluate the effect of thyroid dysfunction in ICU patients and their need for mechanical ventilation (MV).

**Methods:**

A single-center, prospective, observational study was conducted on patients admitted to medical and surgical ICU between 2013-2014.. Clinical and paraclinical findings (free triiodothyronine, free thyroxine and thyroid stimulating hormone) were documented for all patients. Regression analysis and chi-square were used for death and MV outcome variables.

**Results:**

We included 502 patients. Of these, 340 (67.7%) were admitted to the medical ICU. Results of thyroid function tests were normal in 320 (64%) and 162 (32.3%) medical and surgical ICU patients, respectively. Euthyroid sick syndrome (ESS) was documented in 86 patients (17%). Mortality was twice higher among surgical ICU patients with ESS compared to those with normal thyroid function (p=0.085), which is not statistically significant. Based on thyroid function status, no differences in the risk to be mechanically ventilated was found between medical or surgical ICU patients.

**Conclusion:**

There is a significant association between ESS and mortality in ICU patients. Future studies should determine whether abnormal thyroid function increases the risk for MV in ICU patients.

## INTRODUCTION

Thyroid hormones play an essential part in human metabolism.^1^ Thyroid hormones support anabolism, intricate in cardiovascular system function (increase heart contractility and cardiac output), and reduce triiodothyronine/reverse triiodothyronine (T3/rT3) ratio, which frequently relates to normal or low thyroid stimulating hormone (TSH) and thyroxine (T4) levels.^2^ Euthyroid sick syndrome (ESS) is considered when patients with non-thyroidal illness (NTI) demonstrate abnormal thyroid function.^2^ Among intensive care unit (ICU) patients, ESS or low-T3 syndrome is more common than true hypothyroidism.^3^,^4^ A variety of changes in critically ill patients have been observed, including low T3 levels, followed by low T4 and TSH levels. Deiodination from T4 to T3 through marginal (hepatic) enzymes (inhibition of 5′- deionidase) leads to a reduction of T3 and proliferation in rT3 that is biologically inactive.^5^

Generally, the severity of ESS is related to the severity of illness in seriously ill patients. Hence, showing a remarkable fall in total T3 and T4 levels; this state is called the low-T3 syndrome or ESS, which has a poor prognosis.^6^ Patients with low or undetectable TSH levels have increased morbidity and mortality.^7^ Some authors have reported a relationship between hypothyroxinemia and mortality in seriously ill patients with sepsis.^8^ More so, it has been suggested that primary hypothyroidism affects respiration by causing abnormalities in the respiratory system;^9^ however, the mechanism underlying the need for mechanical ventilation (MV) in patients with ESS is unclear.^10^

The objective of this study was to assess thyroid function in medical and surgical ICU patients and to determine the relationship between thyroid function and clinical outcomes as well as mortality. Furthermore, it aimed to evaluate the effect of thyroid dysfunction on the need for MV.

## METHODS

A single-center, prospective, observational, cross-sectional study was conducted on patients admitted to the medical and surgical ICU of King Abdulaziz University Hospital, Jeddah between 2013 and 2014. Patients were included provided they were critically ill and admitted to the ICU, irrespective of their gender, race, ethnic group and age. We excluded all patients who were taking thyroid hormone preparations or anti-thyroid medications. The study was approved by the Institutional Ethics Committee at King Abdulaziz University.

The medical history of patients, mortality, need for mechanical ventilation, and the length of stay at ICU was documented. Venous blood samples for thyroid function tests (TSH, FT3, and FT4) were collected on the first to third days after ICU admission. Based on the results of thyroid function tests, patients were considered to be euthyroid, hypothyroid, hyperthyroid or to have ESS based on the following definitions:^11^

- Euthyroidism was defined as a normal TSH level (0.45 - 4.50 mU/L), with FT4 and FT3 within the normal ranges.
- Hypothyroidism was defined as a TSH concentration ≥ 10 mU/L and FT4 level < 0.70 ng/dL.
- Hyperthyroidism was characterized as a TSH concentration < 0.10 mU/L, with an elevated FT4 level.
- Euthyroid sick syndrome was characterized as low FT4, FT3 and TSH levels.

### Statistical analysis

To describe the patients’ baseline characteristics, the proportion for dichotomous variables as well as their means and standard deviations were measured. Mortality and MV outcome were compared between groups using the chi-square test and regression analysis. Multiple logistic regression analysis was performed to control for potential confounding variables (age, sex, surgery prior to ICU admission, and acute renal failure). To test for statistical significance, a 95% confidence interval was estimated. Because of non-normal distribution, length of stay at the ICU was estimated among survivors using medians and interquartile ranges. All analyses were performed using STATA (Data Analysis and Statistic Software, Texas, USA), version 12.

## RESULTS

### Baseline Characteristics

We enrolled 502 consecutive patients. Of these, 340 (67.7%) were admitted to the medical ICU (Table-I). The results of thyroid function tests were normal in 320 (64.0%) and 162 (32.3%) patients admitted to the medical and surgical ICU, respectively. Euthyroid sick syndrome was documented in 86 ICU patients (17%); 16% were medical ICU patients, while 19.3% were surgical ICU cases. 51.2% of surgical ICU patients had sepsis. Other characteristics of the patients are as shown in Table-I.

**Table-I:** Baseline characteristics of the patients stratified by thyroid function status^1^.

Characteristics	Normal (n=320)	Hypothyroidism (n= 75)	Hyperthyroidism (n=21)	ESS (n=86)
Medical Patients	(n=219)	(n=49)	(n=17)	(n=55)
Mean (SD) age (years)	56 (17.6)	58.1 (19.1)	59.9 (19.2)	56.2 (19.9)
Males	97 (44.3)	20 (40.8)	9 (52.9)	26 (47.3)
ARF	36 (16.5)	9 (18.4)	0 (0.0)	8 (15.4)
Surgical Patients*	(n=101)	(n=26)	(n=4)	(n=31)
Mean (SD) age (years)	53.3 (18.6)	59.1 (15.9)	52 (19.1)	57.1 (15.6)
Males	49 (48.5)	12 (46.2)	0 (0.0)	11 (35.5)
ARF	11 (10.9)	4 (16.0)	0 (0.0)	5 (16.1)
Sepsis	48 (47.5)	12 (46.2)	2 (50.0)	16 (51.6)

*Abbreviations:* ARF, acute renal failure; ESS, euthyroid sick syndrome; SD, standard deviation.

1 Data are presented as frequency (percent) unless otherwise stated.

* Surgical procedure prior to critical care admission.

### Mortality

**Fig.1:**
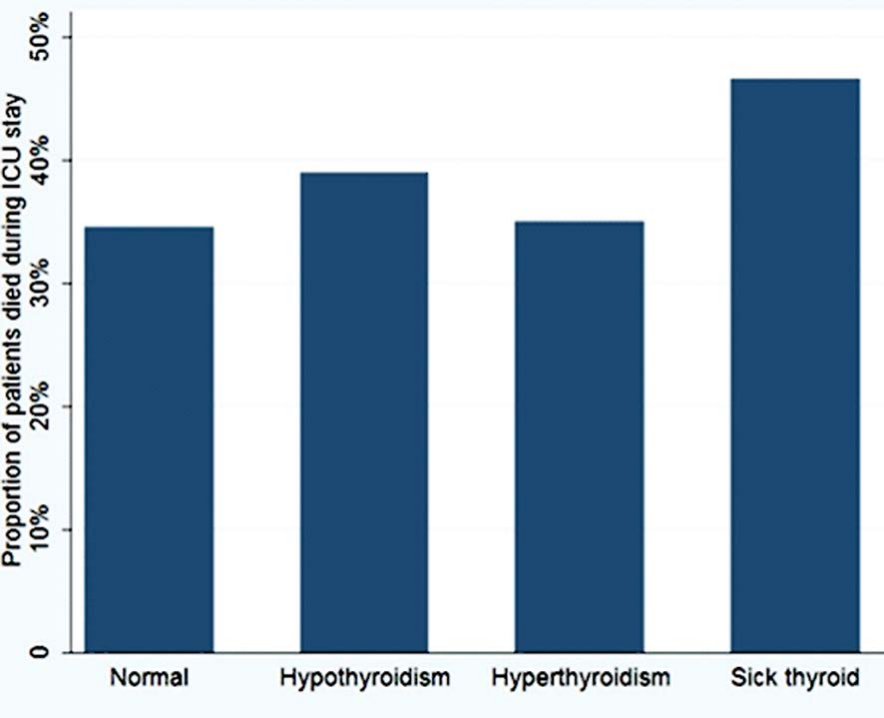
Mortality during intensive care unit stay of the patients stratified by thyroid function status.

The highest mortality occurred among patients with ESS (Fig.1). Patients with hyperthyroidism had a lower risk than those with normal thyroid function; however, these results were not significant (Table-II). Similarly, no difference in mortality was found between medical and surgical ICU patients based on thyroid function status with p value of 0.75 and 0.082 consecutively.

**Table-II:** Mortality during intensive care unit stay stratified by thyroid function status.

	Frequency (%)	Crude OR (95% CI)	Adjusted OR^1^ (95% CI)	P-Value
*Medical Patients*				
Normal (n=212)	82 (38.7)	Reference	Reference	0.72
Hypothyroidism (n=46)	18 (39.1)	1.02 (0.53, 1.96)	0.94 (0.48, 1.84)	
Hyperthyroidism (n=16)	5 (31.3)	0.72 (0.24, 2.15)	0.71 (0.23, 2.18)	
ESS (n=55)	25 (45.5)	1.32 (0.73, 2.40)	1.39 (0.74, 2.62)	
*Surgical Patients*					
Normal (n=101)	26 (25.7)	Reference	Reference	0.085
Hypothyroidism (n=26)	10 (38.5)	1.8 (0.73, 4.50)	1.6 (0.59, 4.10)	
Hyperthyroidism (n=4)	2 (50)	2.9 (0.39, 21.5)	4.6 (0.55, 39)	
ESS (n=31)	15 (48.4)	2.7 (1.2, 6.2)	2.6 (1.1, 6.4)	

*Abbreviations:* CI, confidence interval; ESS, euthyroid sick syndrome; OR, odds ratio.

1 Adjusted for age, sex, and acute renal failure.

### Need for mechanical ventilation based on thyroid function status

**Fig.2:**
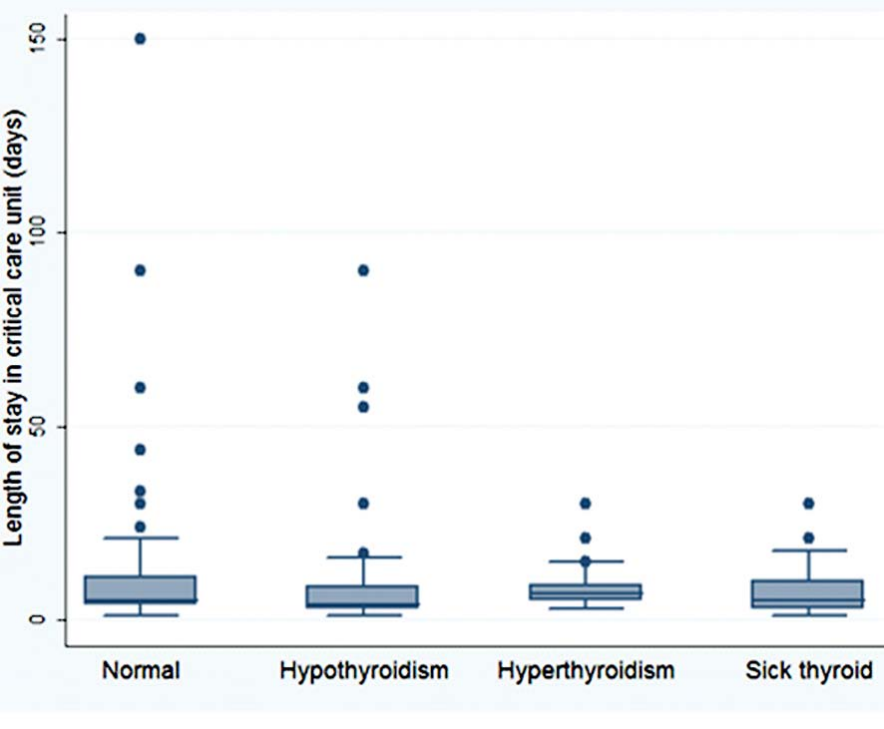
Median length of intensive care unit stay among survivors was five days (range, 4.0-11.0 days) for patients euthyroid patients, four days (range, 3.0-8.8 days) for hypothyroid patients, seven days (range, 4.5-12.0 days) for hyperthyroid patients, and five days (3.0-11.0 days) cases with euthyroid sick syndrome.

After adjusting for age, sex, and acute renal failure, logistic regression analysis demonstrated that among medical ICU cases, hypothyroid (OR, 1.38; 95% CI, 0.72-2.64), hyperthyroid (OR, 0.71; 95% CI, 0.23-2.18) and ESS patients (OR, 1.39; 95% CI, 0.74-2.62) had a higher risk than euthyroid patients to be mechanically ventilated (P-value=0.75). No difference in the risk of being mechanically ventilated was observed among surgical ICU patients based on their thyroid status function. The ICU length of stay was longest for hyperthyroid patients, while those with hypothyroidism had the shortest stay (Fig.2).

## DISCUSSION

Our study showed that 64.0% and 32.3% patients were admitted to the medical and surgical ICU, respectively. Euthyroid sick syndrome was documented in 16% of medical ICU patients compared with the 19.3% of surgical ICU cases. Further, approximately half of surgical ICU patients had sepsis. Sepsis is considered the leading cause of death in non-cardiac ICU patients.^5^ The National Surgical Quality Improvement Program database, in a recent analysis has reported that sepsis and septic shock caused a ten-fold increase in mortality and morbidity than preoperative myocardial infarction and pulmonary embolism in the surgical population.^12^ In another study, acute renal failure and sepsis were reported as a common cause of death in medical and surgical ICU patients.^13^

**Table-III:** Need for mechanical ventilation among intensive care unit patients stratified by thyroid function status.

	Frequency (%)	Crude OR (95% CI)	Adjust. OR* (95% CI)	P-Value
*Medical Patients*				
Normal (n=219)	120 (54.8)	Reference	Reference	0.75
Hypothyroidism (n=49)	31 (63.3)	1.4 (0.75, 2.7)	1.38 (0.72, 2.64)	
Hyperthyroidism (n=17)	10 (58.8)	1.17 (0.43, 3.2)	1.19 (0.43, 3.3)	
Sick thyroid (n=54)	31 (57.4)	1.1 (0.61, 2.0)	1.14 (0.61, 2.12)	
*Surgical Patients*					
Normal (n=101)	47 (46.5)	Reference	Reference	0.608
Hypothyroidism (n=26)	12 (46.2)	0.98 (0.41, 2.3)	1.01 (0.41, 2.5)	
Hyperthyroidism (n=4)	3 (75)	3.4 (0.35, 34.3)	4.4 (0.43, 44.9)	
ESS (n=31)	17 (54.8)	1.4 (0.62, 3.1)	1.38 (0.6, 3.2)	

*Abbreviations:* CI, confidence interval; ESS, euthyroid sick syndrome; OR, odds ratio.

^1^Adjusted for age, sex, and acute renal failure.

Our study demonstrates an association between ESS and ICU. In this report, it was mainly associated with sepsis in surgical ICU patients. Many studies conducted on ICU patients have showed that low T3 was a good predictor of mortality.^14^ A similar finding was also observed in our study, where mortality was twice higher among surgical ICU patients with ESS as compared with patients who had normal thyroid function.

Peeters *et al.*,^15^ in a study conducted on 79 critically ill patients, reported that serum iodothyronine levels were low in the muscle and the liver. This suggests that a reduction in T3 and T4 levels during a critical illness occurs in the tissues. Which could explain the higher mortality among ESS.^15^,^16^ Thorough serum thyroid hormone investigation may be required to differentiate ESS from either hypothyroidism or hyperthyroidism in critically ill patients suspected of having abnormal thyroid dysfunction.^17^-^19^

Hypothyroidism is associated with respiratory failure, and it is a cause of ventilator dependence. Impairment of normal ventilator responses to hypercapnia and hypoxia, diaphragmatic and skeletal muscle dysfunction, pleural effusions, and obstructive sleep apnea are assumed to be the major causes of respiratory failure in hypothyroidism.^20^ Correction of hypothyroidism was reported to be beneficial in weaning these patients from MV.^21^,^22^ In our cohort, hyperthyroid patients required MV than other patients with thyroid dysfunction. It is possible that ventilator dependence was higher in this group because of these patients had sepsis.

Intensive care unit length of stay varied among our patients: 1-160 days for medical ICU and 1-90 days for surgical ICU cases. However, the duration and length of stay among survivors in the medical or surgical ICU did not correlate with thyroid function status. Thus, an important question that arises from our observations is whether T3/T4 supplementation can improve survival in critically ill patients.

Several studies identify low T3 without increased TSH as an adaptive reaction (metabolically protective), and T3 or T4 measurement is therefore not required in these cases.^23^,^24^ Furthermore, reduced deiodinase activity in ESS could hamper marginal conversion of T4 to T3. The therapeutic role of thyroid hormones in controlling ESS is still unclear and anticipates additional controlled randomized trials.^23^,^24^

### This study has several limitations

Results of thyroid function investigations may have been affected by the use of medications (for example, propranolol, benzodiazepines, furosemide and dopamine). As the use of these medications promote imbalance in thyroid hormone levels, it is difficult to adjust probable confounders in clinical trials and practice. However, we tried to avoid the effect of medication on thyroid function tests by extracting blood from patients at the time of admission and ensured that they were not taking drugs that could affect thyroid function like thyroxine replacement or anti-thyroid medications and other medications. The duration of MV was not reported in our study.

Overall, this study shows that there is an association between ESS and ICU mortality. A major risk factor for PMV in mechanically ventilated, critically ill patients may be attributed to thyroid function dysfunction (ESS, hypothyroidism and hyperthyroidism). It is still unknown whether these are biochemical prognostic markers or whether they actually contribute to the development and progression of respiratory failure. By assessing potential benefits in the respiratory functions of critically ill patients after a replacement treatment, clinicians may find an answer to control thyroid dysfunction in ICU patients.
